# The human brain in space: a meta-analysis of neuroimaging evidence

**DOI:** 10.3389/fpsyg.2026.1748118

**Published:** 2026-04-30

**Authors:** Scaramelli Anna, Seghezzi Silvia, Elisa Raffaella Ferre

**Affiliations:** Birkbeck College, University of London, London, United Kingdom

**Keywords:** altered gravity, brain connectivity, fMRI, galvanic vestibular stimulation, neuroimaging, parabolic flight, space analogs, spaceflight

## Abstract

**Introduction:**

Human space exploration is progressing into an unprecedented era characterized by extended-duration missions, the establishment of permanent lunar bases, and planned crewed voyages to Mars. These activities introduce important physiological challenges, primarily driven by exposure to altered gravity environments. Microgravity disrupts vestibular input, generating sensory conflicts that impair spatial orientation, motor coordination, and cognitive performance. Although adaptation to such conditions involves neuroplasticity, the precise neural mechanisms underlying altered gravity exposure remain unclear.

**Methods:**

To address this knowledge gap, we performed a coordinate-based meta-analysis of 15 neuroimaging studies examining functional brain changes associated with spaceflight and validated ground-based analogs. Activation likelihood estimation (ALE) was used to identify convergent patterns of brain activity across studies.

**Results:**

The analysis revealed a predominantly right-lateralized network centred on primary sensorimotor cortices, including the precentral and postcentral gyri, as well as the insula and opercular cortex.

**Discussion:**

These findings suggest that alterations in brain dynamics reflect neuroplastic adaptations to the absence or modification of gravitational signals, supporting the recalibration of internal models that predict and compensate for gravity’s influence on perception and motor behaviour.

## Introduction

Since Neil Armstrong first stepped onto the lunar surface, human space exploration has captured the imagination of scientists, policymakers, and the public alike. What began as a race between nations has evolved into a global scientific and technological pursuit, pushing the boundaries of what humans can achieve beyond Earth. Today, we are witnessing a new phase in space exploration: one marked by plans for long-duration missions, permanent lunar bases, commercial spaceflight, and eventual crewed missions to Mars. These developments are extending humanity’s reach beyond Earth while presenting increasingly complex challenges for human physiological resilience, adaptation and survival. Crews embarking on such missions face multiple environmental hazards, with gravitational variation among the most critical. These range from prolonged microgravity, brief periods of hypergravity during launch and landing, to partial gravity on the Moon or other planetary bodies. Other stressors include extended isolation and confinement in restricted habitats, exposure to space radiation, life in a closed and potentially hostile spacecraft environment, and the operational demands of extreme distance from Earth. A central concern is how the human brain adapts to conditions of reduced or absent gravity. Gravity has shaped life on Earth for billions of years, influencing the evolution of sensory, motor and cognitive systems. As missions grow longer and more frequent, understanding neural adaptation to altered gravity will be critical for astronaut health, performance and mission success.

Since the origin of life, all organisms have evolved under Earth’s gravitational acceleration of 9.81 m/s^2^, commonly referred to as 1 g. Gravity is arguably the most constant and pervasive environmental feature, influencing biological development, physiology and behavior. In humans, when the head moves relative to gravity, the otolith organs in the vestibular system detect this change. This mechanical stimulation is transduced into neural signals that relay crucial information about the magnitude and direction of gravitational forces to the central nervous system. Vestibular input begins early in fetal development and continues to provide sensory information throughout life. The vestibular system is distinguished by its unique neuroanatomical organization. Unlike other sensory modalities, no single, unimodal primary vestibular cortex has been identified in the mammalian brain (Angelaki and Cullen, 2008). Instead, vestibular signals are processed across a widespread network of cortical and subcortical regions. Electrophysiological studies in non-human primates have pinpointed the Parieto-Insular Vestibular Cortex (PIVC) as a central hub within this distributed vestibular network (Guldin and Grüsser, 1998). In humans, functional neuroimaging studies have demonstrated that the homologous vestibular network involves multiple cortical areas, including the posterior parietal operculum, secondary somatosensory cortex, inferior parietal cortex, superior temporal cortex, posterior insula, and premotor cortex (Bottini et al., 1994, 1995; Dieterich and Brandt, 2015; Eickhoff et al., 2006; Zu Eulenburg et al., 2012). Vestibular information is integrated with visual, somatosensory, proprioceptive, and visceral inputs within this widespread multisensory network. These vestibular-multisensory processes enable the brain to generate coherent spatial representations necessary for effective sensorimotor function and environmental interaction.

Humans are exceptionally adapted to Earth’s gravity. For instance, we can accurately predict the acceleration of falling objects even with incomplete visual information (Zago et al., 2004), and our motor behaviors, such as reaching, grasping, and catching, are finely tuned to counteract terrestrial gravitational force (Lacquaniti et al., 2013; Monache et al., 2019). Such abilities demonstrate that gravity is not merely detected, but is deeply *embedded* within the neural computations underlying both perception and action. Accordingly, the physical constraints imposed by terrestrial gravity are internalized by the brain, forming what is known as the *Internal Model of Gravity*. This model reflects a dynamic neural representation of gravity, supporting the brain’s ability to generate predictions for perceptual and motor functions (Lacquaniti et al., 2013; Zago and Lacquaniti, 2005). Operating under Bayesian principles, the internal model integrates prior experience with real-time sensory input to minimize prediction error and guide adaptive behavior (Lackner and DiZio, 2005; Jörges and López-Moliner, 2017). Neuroimaging studies suggest this model is encoded in a network of regions including the posterior parietal cortex, cerebellum, temporo-parietal junction, insula, supplementary motor area, and somatosensory cortex (Indovina et al., 2005; Zu Eulenburg et al., 2012; Raiser et al., 2020). Importantly, this network substantially overlaps with the distributed vestibular projections, underscoring the critical role of vestibular processing in the neural representation of gravity.

Under normal terrestrial conditions, the otolith organs detect gravitoinertial acceleration by modulating their neural output patterns, providing the brain with continuous information about head position relative to gravity. In microgravity, however, the vestibular otoliths are effectively unloaded and lose their ability to signal static head orientation. As a result, during spaceflight the central nervous system tends to interpret all otolith activity as indicative of linear head translation rather than tilt. Perception and motor control rely on the brain’s ability to integrate information from multiple sensory modalities, including vestibular, visual, proprioceptive, and somatosensory systems. This process involves comparing actual sensory inputs against predicted signals generated by internal models. Alterations in gravitational forces disrupt vestibular input, thereby challenging this comparison process and leading to sensory conflict and impairments in spatial orientation, motion estimation, and motor coordination (Reschke et al., 2017; Clément et al., 2020). Adaptation occurs, but it is often partial, varies across individuals, and may not fully compensate for the altered gravity environment (White et al., 2020; Barra et al., 2010). MRI studies have reported macrostructural alterations such as upward brain shift and narrowing of the central sulcus (Roberts et al., 2017; Lee et al., 2019), as well as microstructural changes including gray matter volume reductions in frontal and temporal regions and increases in sensorimotor areas (Koppelmans et al., 2016; Hupfeld et al., 2020). White matter changes have been identified in pathways involved in vestibular processing (Doroshin et al., 2022) and increased ventricular volume has been linked to cerebrospinal fluid shifts and intracranial pressure changes (Van Ombergen et al., 2019; Kramer et al., 2020). Functional neuroimaging has revealed alterations in resting-state connectivity, with reductions observed in vestibular and sensorimotor networks and increased coupling in visual and proprioceptive systems (Pechenkova et al., 2019; Hupfeld et al., 2022). Changes in connectivity patterns have also been reported between the cerebellum, motor cortex, and default mode network (Demertzi et al., 2016; Van Ombergen et al., 2017a), as well as deactivation in somatosensory and visual cortices (Pechenkova et al., 2019).

While animal studies investigating nervous system responses to spaceflight and microgravity date back several decades, investigations of human brain structural and functional changes are comparatively recent, with early groundwork established by Roberts et al. (2010) and expanded upon in subsequent years (Wuyts et al., 2025). Progress in this field has been limited by small sample sizes, heterogeneous and often non-standardized experimental methodologies, and inconsistent data reporting across studies (Arshad and Ferré, 2023). Consequently, a systematic synthesis of how altered gravity exposure affects the human brain across diverse paradigms is still lacking. To address this critical knowledge gap, we conducted a meta-analysis of functional MRI studies encompassing both actual spaceflight and validated ground-based analog models, which replicate key aspects of microgravity within controlled experimental settings, allowing for more rigorous and comparable assessment of neural adaptations.

Brain structural and functional changes have been reported across a range of altered gravity conditions, including actual spaceflight, head-down bed rest (HDBR), HDBR combined with elevated CO₂, parabolic flight and galvanic vestibular stimulation, Head-Down Bed Rest (HDBR) involves maintaining participants in a 6° head-down tilt position for extended periods, typically ranging from days to weeks. This posture induces a cephalad fluid shift similar to that experienced in microgravity, along with vestibular unloading due to reduced head movement relative to gravity, effectively simulating sensorimotor changes observed in astronauts (Hargens and Vico, 2016). Parabolic Flight exposes subjects to a series of parabolas flown by specially equipped aircraft, creating brief (20 s) alternating phases of microgravity (0 g) and hypergravity (1.8 g). This transient exposure enables real-time assessment of neural responses to rapid gravitational transitions, mimicking the dynamic gravity environment during launch and re-entry (Shelhamer, 2016). Galvanic Vestibular Stimulation (GVS) involves delivering low-intensity electrical currents to the mastoid processes, selectively perturbing vestibular afferents and artificially inducing sensations of imbalance and self-motion. By manipulating vestibular inputs without physical changes in gravity, GVS models the sensorimotor conflicts and vestibular dysfunctions characteristic of weightlessness (Moore et al., 2011). These analogs reliably reproduce many of the neurophysiological and behavioral changes seen in astronauts, such as structural brain shifts, alterations in functional connectivity, and impairments in postural control and spatial orientation (Koppelmans et al., 2016; Cassady et al., 2016; Lee et al., 2021). By integrating data across these domains, the present meta-analysis aims to identify convergent patterns of altered brain activity and connectivity induced by microgravity exposure.

## Materials and methods

### Data collection and preparation

This meta-analysis was conducted and reported in accordance with the Preferred Reporting Items for Systematic Reviews and Meta-Analyses (PRISMA) 2020 guidelines (Page et al., 2021). We searched the Pubmed database^1^ in March 2026, by using the following search strings: “gravity” AND (“neuroimaging” OR “fMRI” OR “resting state”); “spaceflight” AND (“neuroimaging” OR “fMRI” OR “resting state”);” space flight” AND (“neuroimaging” OR “fMRI” OR “resting state”); “microgravity” AND (“neuroimaging” OR “fMRI” OR “resting state”); “GVS” AND (“neuroimaging” OR “fMRI” OR “resting state”); “CVS” AND (“neuroimaging” OR “fMRI” OR “resting state”); “vestibular stimulation” AND (“neuroimaging” OR “fMRI” OR “resting state”).

We carefully reviewed the resulting manuscripts and included in the current meta-analysis only those studies that met the following criteria:

- Included healthy adult participants;
- Used fMRI (resting-state or task-based);
- Reported whole-brain analyses (i.e., not limited to partial brain coverage or region-of-interest approaches);
- Provided activation coordinates in standard space (either Montreal Neurological Institute [MNI] or Talairach [TAL]; TAL coordinates were converted to MNI using GingerALE);
- Included either the main effect of altered gravity (GVS vs. Rest, noisy Galvanic Vestibular Stimulation (nGVS) vs. Rest, GVS vs. Rest, nGVS vs. GVS) or the contrast between pre and post exposure to gravity alteration (Pre vs. Post Space Flight, Pre vs. Post HDBR, Pre vs. Post Parabolic Flight, Pre vs. Post HDBR+ CO₂)
- Published in peer-reviewed journals.

We retried 375 entries. 265 studies were excluded by inspecting the title. Further 87 studies were rejected based on the abstract inspection. Finally, 8 studies were excluded by the full manuscript inspection (Supplementary Figure S1). The final dataset included 15 studies (Supplementary Table S1) that investigated functional brain changes related to altered gravity. In the selected studies, conditions of microgravity exposure where caused by spaceflight (Demertzi et al., 2016; Hupfeld et al., 2022; Jillings et al., 2023; Salazar et al., 2023) and space analogs, specifically HDBR (Cassady et al., 2016; Liao et al., 2012; Liao et al., 2013; Liao et al., 2015; Tays et al., 2024; Yuan et al., 2018); HDBR+CO₂ (Hupfeld et al., 2020; McGregor et al., 2021); GVS (Mitsutake et al., 2020; Mitsutake et al., 2021) and Parabolic Flight (Van Ombergen et al., 2017b). The studies considered included data from various fMRI paradigms assessing the effects of gravity alteration. These comprised resting-state fMRI comparisons before and after gravity changes (Cassady et al., 2016; Demertzi et al., 2016; Jillings et al., 2023; Liao et al., 2012, 2013, 2015; McGregor et al., 2021; Van Ombergen et al., 2017b); task-based fMRI involving spatial working memory (SWM) tasks performed pre- and post-gravity alteration (Salazar et al., 2023); and fMRI studies comparing pre- and post-gravity alteration with concurrent vestibular stimulation during scanning sessions (Mitsutake et al., 2020, 2021; Tays et al., 2024; Yuan et al., 2018). These studies focused on either univariate activations (Hupfeld et al., 2020; Hupfeld et al., 2022; Liao et al., 2012; Liao et al., 2015; Mitsutake et al., 2020; Mitsutake et al., 2021; Tays et al., 2024; Yuan et al., 2018) or whole-brain brain connectivity (Cassady et al., 2016; Demertzi et al., 2016; Jillings et al., 2023; Liao et al., 2013; McGregor et al., 2021; Salazar et al., 2023; Van Ombergen et al., 2017b). The number of available studies was insufficient to support separate meta-analyses for activations and connectivity. Therefore, we combined both modalities in a single meta-analysis, an approach aiming to provide a comprehensive overview of functional brain changes related to altered gravity.

### Activation likelihood estimation (ALE) analysis

Coordinates from 22 experiments (94 foci, *N* = 377 subjects) were analysed using GingerALE v3.0.2 (Eickhoff et al., 2009; Turkeltaub et al., 2012). The analysis employed the Turkeltaub non-additive method (Turkeltaub et al., 2012). Statistical significance was determined through cluster-level inference (1,000 permutations), using a cluster-forming threshold of *p* < 0.05 (uncorrected) followed by cluster-level family-wise error (FWE) correction at *p* < 0.05.

## Results

**Figure 1:**
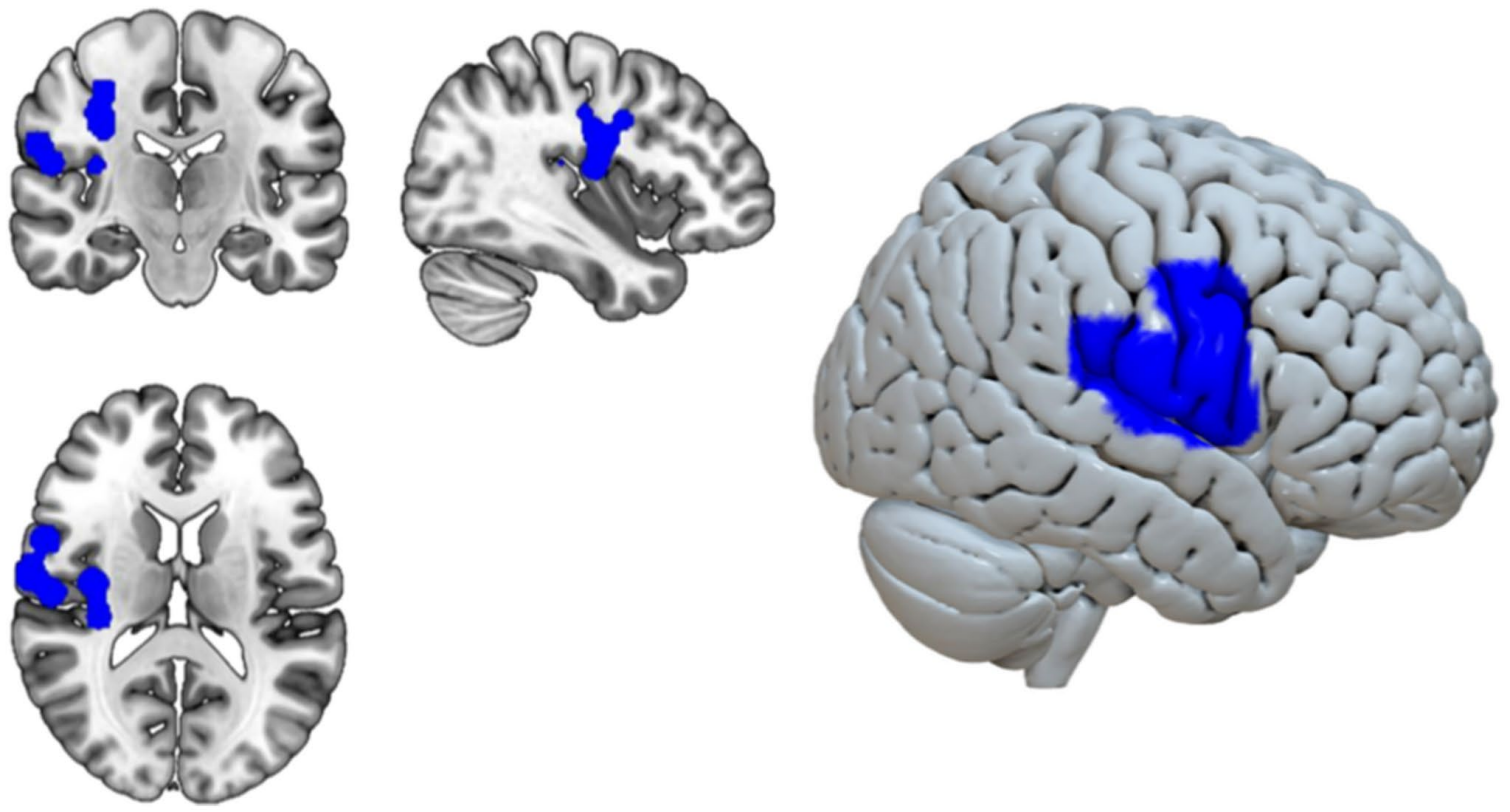
Results of the ALE meta-analysis showing a significant right-lateralized convergence cluster associated with altered gravity exposure. Blue regions indicate voxels with statistically significant convergence (cluster-level FWE-corrected *p* < 0.05). The cluster spans the precentral and postcentral gyri, insula, and opercular cortex. Images are shown in standard MNI space on coronal, sagittal, and axial views, as well as on a rendered 3D surface.

The ALE meta-analysis identified one significant cluster (18,968 mm^3^, FWE-corrected p < 0.05) with 12 peaks of significant convergence in right-hemisphere regions. The highest convergence occurred in the precentral gyrus [MNI (54, 2, 34); ALE = 0.0172], followed by the postcentral gyrus [(42, −8, 26); ALE = 0.0136]. Additional peaks were located in the postcentral gyrus [(62, −8, 16); ALE = 0.0121], precentral gyrus [(34, −16, 36); ALE = 0.0097], insula [(36, −12, 14); ALE = 0.0090], and opercular cortex [4 foci between (54, −16, 16) and (60, −20, 24); mean ALE = 0.0088 ± 0.0005]. The cluster extended from frontal to temporal regions, with 100% of its volume in the right hemisphere (60.4% frontal lobe, 23.1% sub-lobar, 12.9% parietal lobe) (see Figure 1 and Table 1).

**Table 1:** Peak coordinates and ALE scores within the significant cluster identified in the meta-analysis.

Anatomical area	MNI coordinates						ALE score	*p* value	*Z* score
	Left hemisphere			Right hemisphere					
	x	y	z	x	y	z			
Precentral gyrus				54	2	34	0.01715486	*p* < 0.0001	4.8326197
Postcentral				42	−8	26	0.01361265	*p* < 0.0002	4.147602
Postcentral				62	-8	16	0.01210323	*p* < 0.0003	3.862368
Precentral gyrus				34	−16	36	0.00966759	*p* < 0.0004	3.4136803
Operculum				56	6	18	0.00937033	*p* < 0.0005	3.368657
Postcentral				44	0	34	0.00933443	*p* < 0.0006	3.3602347
Insula				36	−12	14	0.00896142	*p* < 0.0007	3.2922196
Operculum				54	−16	16	0.00890655	*p* < 0.0008	3.2824345
Operculum				60	−20	24	0.00878202	*p* < 0.0009	3.2185977
Precentral gyrus				32	−16	46	0.00878141	*p* < 0.0010	3.2185977
Operculum				58	8	10	0.00835882	*p* < 0.0011	3.0902603
Herschl gyrus				34	−26	16	0.00737404	*p* = 0.002	2.8157544

All peaks are in the right hemisphere. Anatomical labels are based on the Automated Anatomical Labeling (AAL) atlas. Coordinates are reported in Montreal Neurological Institute (MNI) space. All listed peaks fall within the same statistically significant cluster (cluster-level FWE-corrected *p* < 0.05).

## Discussion

Spaceflight presents unique challenges to human physiology, with altered gravity environments posing significant demands on the brain’s ability to maintain sensorimotor and cognitive functions. Understanding how the brain responds and adjusts to these challenging conditions is crucial for the success and safety of future space missions. Here we synthesized neuroimaging findings from 15 studies to characterize consistent patterns of functional brain changes associated with altered gravity exposure under conditions of spaceflight and ground-based analogs (HDBR, parabolic flight, GVS). Our ALE analysis incorporated data from studies reporting both increases and decreases in brain activity and connectivity under altered gravity conditions. This revealed a consistent right-hemisphere cluster of convergence involving key sensorimotor areas - the precentral and postcentral gyri - as well as the insula and opercular cortex. These findings highlight brain’s adaptive responses to changes in gravity, particularly within the vestibular network, comprising regions essential for sensorimotor integration, spatial orientation, and multisensory processing.

The most significant convergence of changes, observed in response to both physical and simulated altered gravity, occurred in the right precentral and postcentral gyri, corresponding to primary motor (M1) and somatosensory (S1) cortices. These regions are critical for sensorimotor function, encompassing voluntary movement planning and execution, the processing of tactile and proprioceptive inputs, and the integration of sensory feedback necessary for accurate motor control. Importantly, vestibular projections have been shown to reach these sensorimotor areas directly, facilitating the integration of vestibular signals with motor and somatosensory processing (Lopez and Blanke, 2011). The alterations observed in these areas suggest that exposure to altered gravitational environments directly affects regions responsible for voluntary movement control and somatosensory processing. The sensorimotor cortex’s role in combining proprioceptive inputs and generating motor commands implies that changes in its activity and connectivity may reflect neuroplastic adaptations to disrupted or unreliable vestibular inputs under altered gravity conditions.

Significant convergence of altered activation and connectivity was also observed in the insula and opercular cortex, underscoring their important role in adapting to changes in gravity. The insula is a key cortical hub within the vestibular network, involved in integrating vestibular signals with visual, somatosensory, and proprioceptive inputs. This multisensory integration supports essential functions such as balance, spatial orientation, self-motion perception, and the maintenance of bodily awareness. Functional neuroimaging studies consistently highlight the insula’s involvement in processing vestibular information and coordinating adaptive responses to changes in head position relative to gravity (Indovina et al., 2005; Zu Eulenburg et al., 2012). Under altered gravity, it likely mediates the brain’s compensatory reweighting of sensory signals, attenuating unreliable vestibular cues while enhancing reliance on visual and somatosensory feedback. Although altered gravity appears to enhance visual dependence when vestibular signals are reduced, the effects on somatosensory processing are less straightforward. For example, Gammeri et al. (2023) reported that somatosensory stimuli were underestimated during HDBR compared to the upright position, suggesting that visual and somatosensory modalities may be differentially influenced by gravitational changes. Furthermore, the insula’s involvement in predictive coding implies that, when faced with conflicting sensory inputs, it updates the brain’s internal model of gravity: a mechanism that is essential for recalibrating perception and guiding action across varying gravitational environments (Lacquaniti et al., 2015).

Consistent alterations were also found in the opercular cortex, likely reflecting its crucial role in sensorimotor remapping during adaptation to altered gravity. Specifically, the vestibular parietal operculum area OP2, a cytoarchitectonically defined subregion within the parietal operculum, serves as a core component of the human vestibular cortex (Eickhoff et al., 2006; Zu Eulenburg et al., 2012). OP2 integrates vestibular, visual, and somatosensory inputs to support perception of self-motion, balance maintenance, and spatial navigation (Lopez and Blanke, 2011) Functionally, this region connects somatosensory and motor areas, enabling rapid motor adjustments by continuously comparing internal models of gravity with incoming sensory information (Lacquaniti et al., 2013; Indovina et al., 2005). This integrative process is essential for facilitating quick and accurate motor corrections in response to changes in gravitational forces.

We observed a marked right-hemisphere dominance in activations and connectivity altered by changes in gravity. This lateralization aligns with a substantial body of research implicating the right hemisphere as a key player in vestibular processing (Indovina et al., 2005; Angelaki and Cullen, 2008). The right hemisphere is often considered specialized for processing global spatial reference frames, which are essential for constructing and maintaining a coherent sense of orientation in space. This specialization likely underpins its heightened sensitivity to gravitational mismatches, as alterations in gravity disrupt the usual sensory signals that inform spatial orientation (Barra et al., 2010; Lopez et al., 2012). Consequently, the asymmetric engagement of the right vestibular network may reflect adaptive plasticity mechanisms aimed at recalibrating spatial representations when confronted with altered gravitational environments. This lateralized plasticity could be critical for maintaining balance, spatial awareness, and sensorimotor integration during exposure to microgravity or other altered gravity conditions. Further, this right-hemisphere predominance resonates with clinical observations where lesions affecting right-hemisphere vestibular areas lead to more pronounced deficits in spatial orientation and perception compared to similar left-hemisphere damage (Dieterich and Brandt, 2015).

Limitations of the present work should be acknowledged. Methodologically, the included studies were characterized by consistently small sample sizes, reflecting the inherent logistical and ethical constraints of research in spaceflight and analog environments. Considerable heterogeneity was observed across studies in gravitational conditions, experimental paradigms, and neuroimaging protocols, which complicates direct comparisons. Specifically, the diversity of gravitational conditions (e.g., actual spaceflight, head-down bed rest, parabolic flight) and the limited number of studies within each condition precluded both the inclusion of gravitational condition as a covariate and the conduct of condition-specific subgroup analyses. In addition, this meta-analysis pooled univariate fMRI activation studies with whole-brain functional connectivity studies, which differ in analytical frameworks and the neural processes they capture; while this approach was necessary given the limited number of studies, it should be considered when interpreting the results. Finally, the literature search was limited to PubMed, and although citation chaining was used to identify additional studies, the absence of a broader multi-database search (e.g., EMBASE, Scopus, Web of Science) may have led to the omission of eligible studies.

The limitations identified in the present work also reflect broader challenges in the study of the human brain under altered gravity. Small sample sizes, methodological heterogeneity, and diverse gravitational conditions are not unique to our meta-analysis but characterize much of the field, limiting comparability and generalization. These challenges invite reflection on how space neuroscience research can be strengthened. Increasing sample sizes through multi-center collaborations or data-sharing initiatives would help overcome the persistent statistical limitations of spaceflight and analog research. Standardizing experimental paradigms and neuroimaging protocols would reduce methodological heterogeneity and improve comparability across studies. Employing complementary neuroimaging and neurophysiological techniques, such as EEG, would not only broaden accessibility for labs with varying resources but also provide temporal resolution to complement fMRI’s spatial precision. Integrating computational modeling could help synthesize findings across heterogeneous datasets and predict neural adaptation to altered gravity. Expanding the methodological toolkit and harmonizing approaches will be essential to overcome current constraints and build a robust, replicable understanding of how the human brain responds to spaceflight and its ground-based analogs.

In conclusion, this meta-analysis synthesizes neuroimaging evidence across multiple spaceflight and ground-based analog studies to reveal consistent brain adaptations to altered gravity. The findings underscore the critical involvement of a right-lateralized network encompassing primary sensorimotor cortices, the insula, and the opercular cortex - regions integral to multisensory integration, spatial orientation, and motor control. Altered activity and connectivity within these areas likely reflect neuroplastic mechanisms that enable the brain to recalibrate internal models of gravity and maintain sensorimotor and cognitive functions in challenging gravitational environments. The prominent right-hemisphere dominance highlights the specialized role of this hemisphere in spatial cognition and vestibular processing, supporting adaptive responses to gravitational mismatches. Understanding these neural adaptations provides crucial insights for optimizing astronaut health and performance during long-duration missions, and informs the development of countermeasures to mitigate the effects of microgravity on the human brain.

## Data Availability

The original contributions presented in the study are included in the article/Supplementary material, further inquiries can be directed to the corresponding author.

## Footnotes

1
www.pubmed.com
