# Supplementary material for: The human brain in space: a meta-analysis of neuroimaging evidence
Source: Front Psychol. 2026 Apr 30;17:1748118. doi: 10.3389/fpsyg.2026.1748118 (PMC13174930; doi:10.3389/fpsyg.2026.1748118)
Supplement: Supplementary file 1 [file Table_1.docx]

| **Author** | **Year** | **Connectivity/ Activity** | **Contrast** | **Contrast Description** | **Task** |
| --- | --- | --- | --- | --- | --- |
| **Spaceflight** | | | | | |
| Demertzi et al. | 2016 | Connectivity | Pre > Post | Decreases in ICC Pre-Post Spaceflight | Resting state fMRI session + mental imagery tasks fMRI session |
| Hupfeld et al. | 2022 | Activity | Pre > Post | Reduction in deactivation Pre-to-Post Spaceflight | Vestibular stimulation via pneumatic skull taps during fMRI |
| Jillings et al. | 2023 | Connectivity | Pre > Post | Decreases in ICC Pre-to-Post Spaceflight | Resting state fMRI |
|  |  |  | Post > Pre | Increases in ICC Pre-to-Post Spaceflight |  |
| Salazar et al. | 2023 | Connectivity | Pre > Post | Decrease in ICC during Spatial working memory task Pre-to-Post Spaceflight | Spatial working memory task during fMRI |
| **HDBR** | | | | | |
| Liao et al. | 2012 | Activity | Pre > Post | Decrease in ALFF Pre-to-Post HDBR | Resting State fMRI |
| Liao et al. | 2013 | Connectivity | Pre > Post | Decreases in Reho Pre-Post HDBR | Resting state fMRI + mental rotation task (outside scanner) |
|  |  |  | Post > Pre | Increases in Reho Pre-Post HDBR |  |
| Liao et al. | 2015 | Activity | Pre > Post | Decreases in ALFF Pre-During HDBR | Resting state fMRI |
|  |  |  | Post > Pre | Increases in ALFF Pre-During HDBR |  |
| Cassady et al. | 2016 | Connectivity | Pre > Post | Decreases in ICC Pre-to-Post HDBR Bed rest | Resting state fMRI |
|  |  |  | Post > Pre | Increases in ICC Pre-to-Post HDBR Bed rest |  |
| Yuan et al. | 2018 | Activity | Post > Pre | Increases in activation Pre-Post HDBR | Vestibular stimulation via skull taps before and after HDBR |
| Tays et al. | 2024 | Activity | Post > Pre | Increase in activation Pre-to-Post HDBR | Vestibular stimulation via pneumatic skull taps during fMRI |
| **HDBR + CO₂** | | | | | |
| Hupfeld et al. | 2020 | Activity | Pre > Post | Increases and decreases in activation during vestibular stimulation across Pre-to-Post HDBR + CO₂ | Vestibular stimulation via pneumatic skull taps during fMRI |
| McGregor et al. | 2021 | Connectivity | Pre > Post | Decrease in ICC Pre-to-Post HDBR+CO₂ | Resting state fMRI + Rod & Frame Test, Functional Mobility Test (outside scanner) |
| **GVS** | | | | | |
| Mitsutake et al. | 2020 | Activity | GVS > Rest | Increases in activation during GVS compared to Rest | GVS during fMRI, followed by postural stability assessment |
| Mitsutake et al. | 2021 | Activity | nGVS > rest | Increase in activation during nGVS compared to Rest | nGVS during fMRI + Resting state fMRI |
|  |  |  | GVS > rest | Increases in activation during GVS compared to Rest | GVS during fMRI + Resting state fMRI |
|  |  |  | nGVS > GVS | Increases in activation during nGVS compared to GVS | nGVS during fMRI + GVS during fMRI |
| **Parabolic Flight** | | | | | |
| Van Ombergen et al. | 2017 | Connectivity | Pre > Post | Increase in ICC Pre-Post Parabolic Flight | Resting state fMRI |
