# Supplementary material for: The human brain in space: a meta-analysis of neuroimaging evidence
Source: Front Psychol. 2026 Apr 30;17:1748118. doi: 10.3389/fpsyg.2026.1748118 (PMC13174930; doi:10.3389/fpsyg.2026.1748118)
Supplement: Supplementary file 2 [file Table_2.DOCX]

Records removed *before screening*:

Duplicate records removed

(n = 73)

Records identified from PubMed:

(n = 448)

**Identification**

Records screened based on title

(n = 375)

Records excluded

(n = 265)

Records screened based on abstract

(n = 110)

Records excluded

(n = 87)

**Screening**

Full text articles inspected

(n = 23)

Records excluded

(n = 8)

Studies included

(n=20

**Included**

**Fig. S1.** PRISMA-style flow diagram showing the literature search and screening process for the activation likelihood estimation (ALE) meta-analysis. The figure details the number of records identified, screened, excluded, and included, along with reasons for exclusion at each stage.
